# Effects of Telerehabilitation on Pain and Disability in Patients with Chronic Neck Pain: A Systematic Review and Meta-Analysis

**DOI:** 10.3390/healthcare12070796

**Published:** 2024-04-06

**Authors:** Geraldine Valenza-Peña, Andrés Calvache-Mateo, Marie Carmen Valenza, María Granados-Santiago, Julia Raya-Benítez, Irene Cabrera-Martos, Esther Díaz-Mohedo

**Affiliations:** 1 Department of Physiotherapy, Faculty of Health Sciences, University of Granada, 60 Av. Ilustración, 18016 Granada, Spain; geraldinevalenza@ugr.es (G.V.-P.); andrescalvache@ugr.es (A.C.-M.); irenecm@ugr.es (I.C.-M.); 2 Department of Nursing, Faculty of Health Sciences, University of Granada, 60 Av. Ilustración, 18016 Granada, Spain; mariagranados@ugr.es (M.G.-S.); juliarb@ugr.es (J.R.-B.); 3 Department of Physiotherapy, University of Málaga, 29071 Málaga, Spain; estherdiaz@uma.es

**Keywords:** telerehabilitation, chronic neck pain, disability, review, meta-analysis

## Abstract

(1) *Background*: This systematic review and meta-analysis explores the effectiveness of telerehabilitation in patients suffering from chronic neck pain, specifically on pain and disability. The research delves into an area of growing significance within the realm of healthcare, aiming to understand the impact of digital interventions on the rehabilitation process for individuals with prolonged neck pain. (2) *Methods*: The comprehensive review encompasses a wide array of studies evaluating the collective outcomes of numerous trials focused on telerehabilitation strategies. In this systematic review, PubMed/MEDLINE, Scopus, and Web of Science databases were systematically searched to identify studies on telerehabilitation’s impact on pain. (3) *Results*: Eight studies met the inclusion criteria. Using the Downs and Black quality assessment, three studies were classified as good and five as fair. The authors identify specific modalities within telerehabilitation, such as remote exercise programs and virtual consultations, that contribute significantly to positive patient outcomes. Meta-analysis indicated a significant overall effect of telerehabilitation on pain reduction (MD = −1.27; 95% CI = −2.06; −0.47; *p* = 0.002). These findings support telerehabilitation’s efficacy in pain management. (4) *Conclusions*: The synthesis of evidence presented in this systematic review and meta-analysis underscores the potential of telerehabilitation as an effective and accessible means of managing chronic neck pain, offering valuable insights for both healthcare practitioners and policymakers in advancing patient-centered care.

## 1. Introduction

Neck pain represents a significant health issue, affecting millions of individuals worldwide and imposing a substantial burden in terms of disability and healthcare costs [1]. A significant number of individuals grappling with neck pain find that a full recovery remains elusive. Among patients experiencing neck pain, a mere 6.3% perceive their discomfort as persistently chronic. This highlights the enduring and often challenging nature of neck pain, underlining the need for comprehensive and sustained approaches to address the diverse factors contributing to its prolonged impact on individuals’ well-being [1]. This disorder, characterized by persistent pain in the cervical region over an extended period, negatively impacts the quality of life and functionality of those affected [2].

Moreover, persistent and/or chronic pain is characterized by its continuous presence for at least three out of the preceding six months. The origins of non-specific neck pain remain elusive, with the onset and recurrence of such pain being acknowledged as multifactorial [3]. While the precise mechanisms initiating pain are not fully understood, certain influences may be modifiable, whereas others may be attributed to personal and environmental factors [4]. These factors encompass aspects such as occupation, headaches, emotional issues, low job satisfaction, sedentary work postures, and a suboptimal physical work environment [5].

The importance of understanding and addressing chronic neck pain lies in its pervasive nature, often leading to disability and reduced quality of life for affected individuals [6]. Persistent neck pain can restrict mobility, hinder daily activities, and contribute to emotional distress [7]. The presence of disability in chronic neck pain underscores the need for comprehensive approaches to pain management, focusing not only on symptom relief but also on enhancing functional capabilities and minimizing the long-term impact on a person’s ability to engage in normal activities [8]. 

It has been previously stated that although pain and disability are interrelated, they should be assessed separately and considered as two distinct aspects of pain [9]. Pain is defined as a subjective experience, and the assessment tools are focused on what the person reports about their pain. These assessment tools include the visual analog scale, which is the most frequently used pain measure, and the numeric rating scale, which allows better discrimination of small changes in pain or pain questionnaires that are reported to be sensitive in detecting health improvement [10]. Regarding disability due to neck pain, the most widely used tool is the neck disability index, published in 1991 [11]. This index has appropriate psychometric properties and has been used in clinical and research settings [12].

In the current landscape of healthcare [13], telerehabilitation has emerged as an innovative strategy to address various chronic health conditions, including neck pain. The convergence of information and communication technology with rehabilitation practices offers new opportunities to deliver effective interventions remotely, overcoming geographical barriers and enhancing access to care [14]. Exploring the potential of telerehabilitation can modify a paradigm shift in how rehabilitation services can be delivered. By leveraging the capabilities of telecommunication technologies [15], healthcare professionals can extend their reach, providing timely and personalized interventions to individuals dealing with chronic neck pain. 

The premise that telerehabilitation [16] can not only provide a convenient approach to rehabilitation service delivery but also has the potential to empower patients by enabling active participation in their recovery process has been in the middle of controversy when applied to chronic pathologies [17]. In this line, telerehabilitation has been defined as a branch of telehealth and is set up as a system for the control or monitoring of remote rehabilitation using telecommunications technologies. The purpose of telerehabilitation is to increase accessibility and improve continuity of care in vulnerable, geographically remote populations with disabilities, with the potential to save time and resources in health care [18]. The detailed exploration of this treatment modality is essential to inform healthcare professionals, patients, and policymakers about its viability and effectiveness in the context of chronic neck pain. The relevance of telerehabilitation in transforming healthcare delivery requires a growing and meaningful body of evidence for its effects that go beyond the traditional boundaries of healthcare [18]. Specifically, the available evidence can elucidate the results of telerehabilitation interventions, offering a comprehensive perspective on their impact on pain management and functional outcomes.

At a time when healthcare is undergoing an accelerated digital transformation, understanding how telerehabilitation can contribute to the successful management of chronic neck pain is crucial for optimizing care, improving patient outcomes, and ensuring accessible and efficient healthcare [19]. However, it has not yet been demonstrated for chronic neck pain. Furthermore, the up-to-date evidence base about the use of telerehabilitation for chronic neck pain rehabilitation has not been reviewed. Therefore, this systematic review and meta-analysis investigate the effects of telerehabilitation in patients with chronic neck pain to improve pain and disability.

## 2. Materials and Methods

### 2.1. Design 

A systematic review and meta-analyses were performed to identify randomized clinical trials reviewing the effects of telerehabilitation on pain and disability in patients with chronic neck pain. The guidelines of the Preferred Reporting Items for Systematic Reviews and Meta-Analyses (PRISMA) were used [20]. The Cochrane Collaboration guidelines for reviewing interventions were also closely followed [21]. We previously registered the protocol of this systematic review on PROSPERO (CRD42023402445).

### 2.2. Search Strategy 

**Figure 1:**
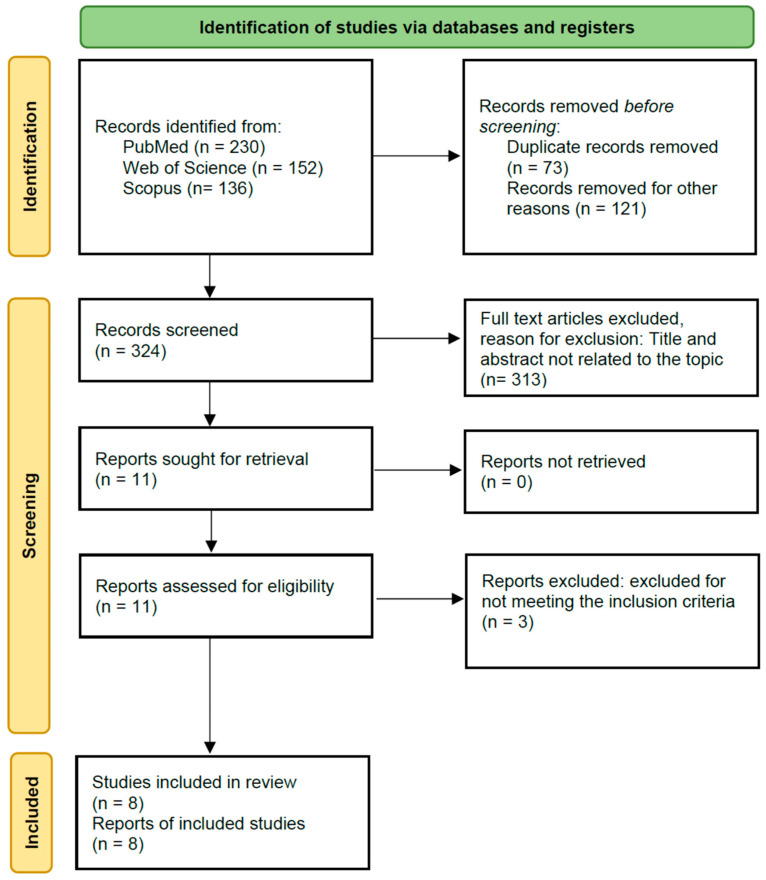
Flow chart of literature search and study selection [20].

A wide search of the literature was conducted for randomized controlled trials indexed on PubMed/MEDLINE, Scopus, and Web of Science databases from their inception to June 2023 in English (Figure 1). The following search strategy was developed for the PubMed/MEDLINE database ((“Telerehabilitation” OR “telerehabilitation program” OR “Telemedicine” OR ”telemedicine program” OR “telehealth” OR “Telehealthcare” OR “telehealth program” OR “telecare” OR “telecare program” OR “electronic health” OR “electronic health program” OR “Virtual Physical Therapy” OR “Tele-physical therapy” OR “home exercise” OR “home exercise program”) AND (“Neck Pain” OR “Chronic Neck Pain” OR “Chronic Pain” OR “Cervical Pain” OR “Cervical Chronic Pain”)). Then, this strategy was adapted to the other databases. Additionally, we screened the reference lists of relevant reviews related to the terms and considered non-English language studies for inclusion if the translation was possible.

### 2.3. Study Selection 

We applied the PICOS (participants, interventions, comparisons, outcome, and study design) model to define the research question. The inclusion criteria were as follows: (1) adult patients with chronic neck pain not related to a traumatic trauma or head and neck cancer; (2) telerehabilitation programs as described by Seron et al. [18]; (3) no intervention or a control intervention without telerehabilitation will be included; (4) pain and disability were the main outcomes, but other pain-related variables will be extracted as secondary outcomes when available; and (5) randomized controlled clinical trials and pilot randomized clinical trials were included.

To reduce potential selection bias, two authors (G.V.-P. and M.C.V.) independently performed the literature search, and the disagreements were resolved by further consultation with a third author (A.C.-M.). The search process included removing duplicates and screening titles, abstracts, and eligible full texts.

### 2.4. Data Extraction 

The following data from the studies included were recorded: author, year of publication, sample size, age (years), gender (percentage of women), disease etiology, and pain characteristics. The full information is summarized in Table 1. Information about the characteristics of interventions containing experimental group interventions, control group interventions, session duration, frequency, program duration, outcome instrument, and main results is summarized in Table 2.

When information was lacking or ambiguous, we tried to contact the study’s corresponding author through email. If data remained unclear or if communication was not possible, we analyzed the available data. The data extraction was independently conducted by two independent reviewers (G.V.-P. and A.C.-M.). 

### 2.5. Methodological Quality of Included Studies 

After obtaining the eligible articles, data extraction and methodological quality assessment were carried out by two independent reviewers (G.V. and A.C.). Methodological quality assessment was evaluated using the Downs and Black Checklist [30], one of the most used methodological quality assessment scales for clinical trials. This tool consists of 27 items, including five subscales, which are as follows: reporting, external validity, internal validity (study bias and confounding), selection bias, and study power. Poor quality is considered when a score of 14 or less is achieved, fair quality between 15 and 19, good between 20 and 25, and excellent quality when the score is higher or equal to 26 [31,32].

### 2.6. Risk of Bias of Included Studies 

The risk of bias for the included randomized controlled trials was assessed using the Cochrane Risk-of-Bias tool version 2.0 (RoB-2) [33]. This tool consists of five domains that focus on the randomization process, deviations from the intended interventions, missing outcome data, measurement of the outcome, and the selection of the reported result. The methodological quality depends on the risk of each of the following subscales: high quality (low risk in all domains), fair quality (high risk in one domain or two unclear domains), and poor quality (two or more unclear domains or there are important limitations that could invalidate the results) [34]. 

### 2.7. Statistical Analysis 

A quantitative synthesis of studies presenting means and standard deviations of pain and disability was carried out using Review Manager (RevMan) software (Version 5.0. The Cochrane Collaboration. Available at revman.cochrane.org). Quantitative data, including the number of patients assessed, mean values, and standard deviations for each treatment arm, was extracted to estimate the overall mean differences between the experimental and control arms. When the studies did not present sufficient data to calculate the effect size (e.g., no means provided, no standard deviation provided), the authors were contacted. We calculated the missing standard deviations when n, *p*-values, or 95% confidence intervals were given via the embedded Review Manager calculator.

We assumed to measure the same underlying symptom or condition, and therefore, standardized mean differences were used as all the scales. The overall mean effect sizes were estimated using random effect models or fixed effect models according to statistical heterogeneity I_2_ tests (for sizes of less than 50%, fixed effect models were used) [35]. We also undertook a visual inspection of the forest plots for outlier studies, explored sources of heterogeneity, and conducted sensitivity analyses by excluding trials that were at a high risk of detection or attrition bias.

## 3. Results

Figure 1 presents the process of the search, screening, and selection of studies. We collected a total of 518 studies from the three electronic databases and 73 duplicate records were removed before screening.

### 3.1. Search Selection

After that, 445 reports were assessed for eligibility. A total of 121 records were excluded as they did not meet the inclusion criteria specified in our study. After screening the titles and abstracts, 313 records unrelated to this review’s topic were also deleted (specifically, population and intervention were not related to the PICOS strategy). Finally, 11 records were full-text screened, and three were excluded due to the control intervention. Finally, eight manuscripts were included in the review [22,23,24,25,26,27,28,29]. 

### 3.2. Characteristics of Studies

The characteristics of the sample and the methodological evaluation of the included studies are shown in Table 1. The studies, published between 2017 and 2023, included randomized clinical trial designs [22,24,25,26,27,28,29] and a pilot randomized trial study [23]. The total sample of patients included in the studies was 689, with a gender distribution in the combined sample of 61.92% female. The mean age of the participants ranged from 22.68 to 60.1 years, with a mean duration of pain reported between 4.02 and 86.4 months. The mean pain intensity reported ranged from 3.97 to 7.3 on a scale of 0–10. These results suggest significant diversity in the demographic and clinical characteristics of the participants included in the studies analyzed.

**Figure 2:**
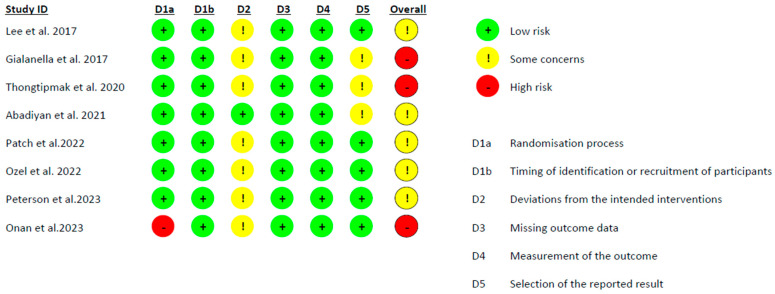
Cochrane Risk-of-Bias tool version 2.0 scores [22,23,24,25,26,27,28,29].

Regarding the methodological quality of the studies evaluated using the Downs and Black quality assessment method, three articles were classified as good [22,25,26], while five were classified as fair [23,24,26,28,29]. Additionally, the risk of bias in all the studies [22,23,24,25,26,27,28,29] was assessed using the RoB-2 tool (Figure 2), which concluded that three of the articles had a high risk of bias [22,24,28], and the remaining had some concerns [23,25,26,27,29]. 

The characteristics of the interventions carried out in the different studies are shown in Table 2. This table includes information about the description of the different interventions, their components, their duration and frequency, the modality, setting, and supervision, as well as the comparator group and the main results found.

The most commonly used interventions are telerehabilitation programs based on therapeutic exercise [22,23,25,26,28,29]. The intervention proposed by Thongtipmak et al. [24] was based on stretching and breathing exercises. In addition, the intervention by Pach et al. [27] consisted of relaxation exercises.

The most frequently repeated telerehabilitation components include tele-education content, symptom, and mood monitoring, as well as physical activity monitoring with personalized feedback to the patient. These elements suggest comprehensive care that addresses both physical and psychosocial aspects of the patient.

The duration and frequency of interventions vary between studies, but on average, interventions last about 8 weeks with a frequency of 4 days per week and a duration of 20 min per session. This indicates consistency in the duration and frequency of interventions that may be optimal for meaningful results.

The most commonly used modality of telerehabilitation intervention is through smartphone apps [23,24,25,27], followed by phone calls [23] and videoconferencing [26,29]. These results suggest a trend toward mobile technology for the delivery of telerehabilitation services. Only Peterson et al. [29] used email as a communication method with patients.

In terms of setting, all the interventions were conducted in the patient’s home [22,23,24,25,26,27,28,29], suggesting significant convenience and accessibility for participants. In addition, most interventions were delivered under supervision [22,23,24,25,26,27,28], either through scheduled calls, videoconferences, or online consultations with healthcare professionals. Only Peterson et al. [29] conducted an unsupervised telerehabilitation program.

The most common comparator group is non-intervention [24,26] or usual care, [27] allowing for an assessment of the specific impact of telerehabilitation interventions compared with standard care. Other studies used a brochure to correct the posture [23], exercise recommendations [22], physiotherapy, postural reeducation [25], and supervised presential exercises as comparator groups [28,29].

Overall, the results suggest that telerehabilitation interventions have a positive effect on reducing pain [22,23,24,25,27,29] and disability [22,23,25,29] compared with control groups. This is evidenced in several studies where the telerehabilitation group showed significant improvement in pain and disability compared with the control group, as indicated by VAS and NDI scores. However, it is important to keep in mind that the results may vary depending on the specific components of the intervention and the study population.

### 3.3. Results Obtained in Meta-Analysis

**Figure 3:**
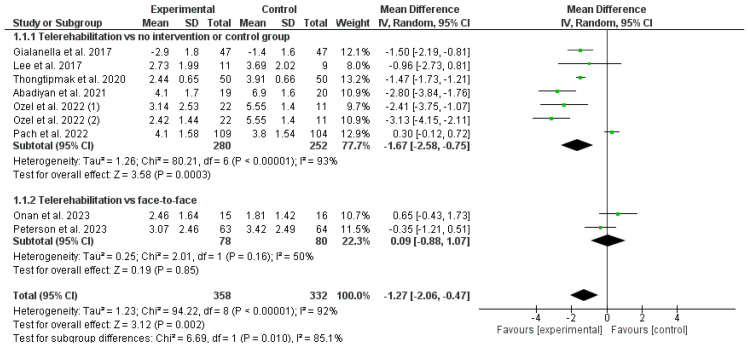
Results of pain [22,23,24,25,26,27,28,29].

The results obtained in the meta-analysis concerning pain were analyzed as shown in Figure 3. The pooled mean difference (MD) showed a significant overall effect of telerehabilitation compared with the comparator groups (MD = −1.27; 95% CI = −2.06; −0.47; *p* = 0.002). The results showed heterogeneity, detecting a significant variability of I_2_ = 92%, not attributable to chance.

A subgroup analysis was carried out. The first subgroup aimed to determine whether telerehabilitation obtained better results than the no-intervention or control group. The pooled MD showed a significant overall effect of telerehabilitation compared with the no-intervention or control groups (MD = −1.67; 95% CI = −2.58; −0.75; *p* = 0.0003). The second subgroup aimed to determine whether performing a treatment through telerehabilitation was not inferior to performing the same treatment in a face-to-face modality. The pooled MD showed a non-significant overall effect of telerehabilitation compared with face-to-face interventions (MD = 0.09; 95% CI = −0.88; 1.07; *p* = 0.85).

**Figure 4:**
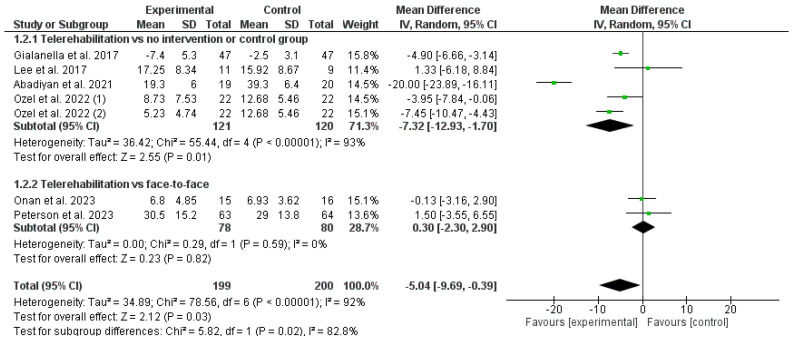
Results of disability [22,23,25,26,28,29].

The results obtained in the meta-analysis concerning disability were analyzed, as shown in Figure 4. The pooled MD showed a significant overall effect of telerehabilitation compared with the comparator groups (MD = −5.04; 95% CI = −9.69; −0.39; *p* = 0.03). The results showed heterogeneity, detecting a significant variability of I_2_ = 92%, not attributable to chance.

A subgroup analysis was carried out. The first subgroup aimed to determine whether telerehabilitation obtained better results than the no-intervention or control group. The pooled MD showed a significant overall effect of telerehabilitation compared with the no-intervention or control groups (MD = −7.32; 95% CI = −12.93; −1.70; *p* = 0.01). The second subgroup aimed to determine whether performing a treatment through telerehabilitation was not inferior to performing the same treatment in a face-to-face modality. The pooled MD showed a non-significant overall effect of telerehabilitation compared with face-to-face interventions (MD = 0.30; 95% CI = −2.30; 2.90; *p* = 0.82).

## 4. Discussion

This systematic review and meta-analysis aimed to investigate the effects of telerehabilitation on pain and disability in patients with chronic neck pain. Our results show positive effects on pain and disability when considering telerehabilitation compared with other interventions. However, our results should be interpreted with caution due to the number of strategies implemented and the dosage of experimental interventions in the studies analyzed.

This systematic review includes eight studies [22,23,24,25,26,27,28,29] that address the effects of telerehabilitation on pain and disability in patients with chronic neck pain. This set of studies provides valuable information on the utility and effectiveness of telerehabilitation in this population, contributing significantly to the current knowledge about treatment options for chronic neck pain.

The results obtained reveal significant findings that have important implications for clinical practice and public health policy. The findings of this review indicate that telerehabilitation interventions have a positive effect on reducing pain and disability associated with chronic neck pain. Specifically, patients who received telerehabilitation interventions were observed to experience a significant decrease in pain intensity and a reduction in disability compared with control groups. These results support the idea that telerehabilitation may be an effective and convenient option for the treatment of chronic neck pain.

In addition, we found that telerehabilitation did not show a significant difference in effectiveness compared with traditional face-to-face interventions. This suggests that telerehabilitation can show no significant differences in its effects from conventional in-person interventions in reducing pain and disability associated with chronic neck pain. A possible reason is the focus on telerehabilitation components. For instance, in the study of Onen et al. [28] the intervention was focused on muscle modifications, and the study of Petersen and Peolsson [29] was focused on self-management skills. Additionally, when comparing face-to-face vs. telerehabilitation programs, the studies included have different components for the intervention and control groups.

Regarding the characteristics of the sample included in the review, it is important to highlight that the selected studies presented considerable variability in terms of the participants’ age, pain duration, and pain intensity. Most of the included studies had a high proportion of women in the sample, which is consistent with the reported prevalence of chronic neck pain in the general population [36]. Compared with other reviews in the field, this sample presents similar heterogeneity in terms of demographic and clinical characteristics, allowing for better interpretation of the results [37,38].

The results of this review are consistent with the existing literature supporting the efficacy of telerehabilitation in a variety of chronic health conditions [39,40,41]. In particular, and due to the high prevalence of this symptom, telerehabilitation is increasingly important in the management of chronic pain [17,42,43]. However, to the authors’ knowledge, this is the first systematic review focused on evaluating the effect of telerehabilitation in the management of patients with chronic neck pain.

If we compare the results of this review with those of other reviews in the field, several consistent trends and findings are observed. First, most of the studies included in this review reported significant improvements in pain and disability in the telerehabilitation group compared with the control group. These findings are in line with previous reviews that have highlighted the potential benefit of telerehabilitation in chronic pain management [17,37,42]. However, it is important to consider that results may vary depending on the specific components of the intervention and the study population. For example, the duration and intensity of the intervention, as well as the participant’s ability to use the technology, may influence the results [44,45].

Concerning disability, the results of this review demonstrate that telerehabilitation has beneficial effects in reducing disability levels in patients with chronic neck pain. These results are in line with those of other reviews previously conducted in other populations [46,47,48,49,50,51,52].

Telerehabilitation interventions were studied and separated according to the different components they offered to patients [51,52]. The most highlighted components among the different interventions included in this systematic review were tele-education content, symptom and mood monitoring, as well as physical activity monitoring with personalized feedback to the patient. These elements suggest comprehensive care that addresses both the physical and psychosocial aspects of the patient. The results obtained in pain and disability in favor of telerehabilitation are positive, but at the same time, we cannot assume the best delivery method or the effects in the mid/long term due to the diversity among studies.

The results of this review have important clinical and public health policy implications. First, they support the feasibility of telerehabilitation as an effective treatment option for chronic neck pain. The ability to perform therapeutic exercises, monitor symptoms, and receive personalized feedback from the comfort of home may significantly improve accessibility and adherence to treatment for this population.

Furthermore, the findings of this study suggest that telerehabilitation may be a comparable alternative to traditional in-person interventions. The lack of a significant difference between the outcomes of telerehabilitation and face-to-face interventions in terms of pain and disability reduction supports the validity and efficacy of this treatment approach. This is particularly relevant in the context of the COVID-19 pandemic, where social constraints have been applied that limit access to in-person health services, leading to increased interest in remote health interventions [53,54,55,56,57].

Despite the promising results, it is important to consider several limitations of this study. First, heterogeneity among the studies included in the review may affect the generalizability of the results. Variability in the intervention methods, duration, and frequency of telerehabilitation may influence the observed effects. In addition, despite the effort to search for and select relevant studies, there is a possibility that some relevant studies may have been omitted due to restrictions in the inclusion criteria or data availability. The exclusion of unpublished studies or studies in languages other than English could also introduce bias into the results. In addition, the duration of follow-up in some studies was limited, making it difficult to assess the long-term sustainability of the effects of telerehabilitation on chronic neck pain.

Considering the limitations identified, further research is needed to consolidate and extend the findings of this study. Future studies could further explore the specific components of telerehabilitation that contribute to pain relief and decreased disability in patients with chronic neck pain. Longitudinal studies evaluating the long-term effects of telerehabilitation on chronic neck pain, as well as investigating patients’ experiences and preferences regarding this treatment approach, would be beneficial.

## 5. Conclusions

In conclusion, this systematic review and meta-analysis show that telerehabilitation is superior to other interventions to improve pain and disability in patients with chronic neck pain. Specifically, the results were significant when compared with the no/control intervention. No significant differences were found when compared with a face-to-face intervention. These results suggest that telerehabilitation may be a useful alternative for patients with chronic neck pain and no access to face-to-face approaches. However, more high-quality research and studies with long-term follow-up are needed to confirm these findings and establish clear guidelines for the implementation of telerehabilitation in clinical practice.

Concerning the clinical implications of this systematic review, telerehabilitation may be an effective and convenient option for the treatment of chronic neck pain, especially in situations where access to in-person medical care is limited. Healthcare professionals should consider integrating telerehabilitation interventions into their clinical practice to improve accessibility and treatment adherence for this patient population. Health policymakers should consider integrating telerehabilitation into healthcare systems to improve access and quality of care for patients with chronic neck pain. Policies and programs that promote the adoption and implementation of telerehabilitation as a viable treatment option in the management of chronic neck pain are needed.

## Figures and Tables

**Table 1:** Characteristics of the studies.

Study (Year)	Study Design/Groups	Sample Size per Group *n* (% Women)	Age Years Mean ± SD	Duration of Pain Months Mean ± SD	Pain Intensity Mean (0–10) ± SD	Downs and Black Score
**Gialanella et al. (2017)** [22]	RCT/2 Groups	**TG:** 47 (89.3) **CG:** 47 (89.3)	**TG:** 56.0 ± 14.0 **CG:** 60.1 ± 11.0	**NR**	**TG:** 6.8 ± 1.3 **CG:** 6.6 ± 1.5	23
**Lee et al. (2017)** [23]	Pilot RCT/2 Groups	**TG:** 11 (55) **CG:** 9 (45)	**TG:** 27.09 ± 4.83 **CG:** 27.56 ± 4.67	**TG:** 50.81 ± 71.72 **CG:** 35.33 ± 18.11	**TG:** 5.20 ± 2.19 **CG:** 4.02 ± 1.75	16
**Thongtipmak et al.** **(2020)** [24]	RCT/2 Groups	**TG:** 50 (82) **CG:** 50 (76)	**TG:** 22.86 ± 1.99 **CG:** 22.68 ± 2.23	**NR**	**TG:** 3.97 ± 0.74 **CG:** 4.06 ± 0.68	18
**Abadiyan et al.** **(2021)** [25]	RCT/3 Groups	**TG:** 20 (50) **CG:** 20 (50)	**TG:** 41.3 ± 8.1 **CG:** 37.4 ± 9.8	**NR**	**TG:** 7.3 ± 0.9 **CG:** 6.4 ± 1.8	21
**Ozel et al. (2022)** [26]	RTC/3 Groups	**TG1:** 22 (72.7) **TG2:** 22 (77.3) **CG:** 22 (59.1)	**TG1:** 36.23 ± 12.45 **TG2:** 34.18 ± 13.03 **CG:** 39.2 7 ± 15.46	**NR**	**TG1:** 6.77 **TG2:** 4.86 **CG:** 5.55	18
**Pach et al. (2022)** [27]	RTC/2 Groups	**TG:** 110 (67.3) **CG:** 110 (71.8)	**TG:** 37.9 ± 11 **CG:** 39.8 ± 11.6	**TG:** 79.2 ± 74.8 **CG:** 86.4 ± 97.7	**TG:** 5.7 ± 1.4 **CG:** 5.8 ± 1.3	20
**Onan et al.** **(2023)** [28]	RCT/2 Groups	**TG:** 15 (73.3) **CG:** 16 (68.7)	**TG:** 37.4 ± 10.58 **CG:** 39.5 ± 10.96	**TG:** 36 **CG:** 60	**TG:** 7.13 ± 1.92 **CG:** 6.75 ± 1.98	16
**Peterson et al. (2023)** [29]	RTC/2 Groups	**TG:** 70 (79) **CG:** 70 (79)	**TG:** 40.4 ± 11.6 **CG:** 40.5 ± 11.4	**TG:** 27.4 ± 21.0 **CG:** 25.2 ± 15.5	**TG:** 5.77 ± 1.87 **CG:** 5.86 ± 1.70	19

SD: standard deviation; RCT: Randomized controlled trial; *n*: number; TG: telehealth group; CG: Control group; NR: not reported.

**Table 2:** Characteristics of the interventions.

Study (Year)	Experimental Intervention Design and Support	Telerehabilitation Components	Intervention Duration and Frequency Weeks Days/Week	Telehealth Setting, and Supervision	Comparator Group	Main Results
**Gialanella et al. (2017)** [22]	Telerehabilitation isolated via phone calls	-Education content -Symptom and mood monitoring -Physical activity monitoring and personalized feedback -Education in self-management skills -Tele-consultation with healthcare professionals -Remote decision support system -Therapeutic exercise program	24 w 5 d/w 20 min	Home Fortnightly scheduled phone calls	Exercise recommendation	Pain (VAS): TG ** > CG ** (*p* < 0.001) Disability (NDI): TG ** > CG ** (*p* < 0.001)
**Lee et al. (2017)** [23]	Telerehabilitation isolated via smartphone app + phone calls	-Education content -Symptom and mood monitoring -Physical activity monitoring and personalized feedback -Therapeutic exercise program	8 w 2 d/w 10–15 min	Work setting Supervised	Brochure to correct the posture	Pain (VAS): TG * > CG (*p* < 0.05) Disability (NDI): TG * > CG (*p* < 0.05) Fear-avoidance belief (FABQ): -Physical activity: TG vs. CG (NSD) -Work: TG vs. CG * (*p* < 0.05) -Health-related quality of life (SF-36): TG vs. CG (NSD)
**Thongtipmak et al. (2020)** [24]	Telerehabilitation isolated via a smartphone app	-Education content -Symptom and mood monitoring -Tele-education in self-management skills -Therapeutic exercise program	15–20 min	Home Supervised	No intervention	Pain (VAS): TG ** > CG * (*p* < 0.001)
**Abadiyan et al. (2021)** [25]	Telerehabilitation via smartphone app combined with a presential exercise program	-Physical activity monitoring and personalized feedback -Therapeutic exercise program	8 w 4 d/w 50 min	Home Supervised	Usual care	TG-CG Pain (VAS): TG > CG *; *p* < 0031 Disability (NDI): TG vs. CG (NSD) Quality of life (SF-36): TG > CG *; *p* < 0.001
**Ozel et al. (2022)** [26]	Telerehabilitation via videoconference	-Physical activity monitoring and personalized feedback -Education in self-management skills -Tele-consultation with healthcare professionals -Therapeutic exercise program	4 w 4 d/w 20 min	Home Bi-weekly individual online sessions	No intervention	TG1 vs. CG: Pain (VAS): TG1 ** > CG (*p* < 0.001) Disability (NDI): TG1 ** vs. CG (NSD) TG2 vs. CG: Pain (VAS): TG2 ** > CG (*p* < 0.001) Disability (NDI): TG2 ** > CG (*p* < 0.001)
**Pach et al. (2022)** [27]	Telerehabilitation via smartphone app	-Symptom and mood monitoring -Education in self-management skills -Therapeutic exercise program	7 d/w 15 min	Home Supervised	Usual care and app for data entry only	Pain intensity (NRS): TG ** > CG (*p* < 0.05) Neck Disability (NDI): TG ** vs. CG (NSD) General and physical health (WHOQOL-BREF): TG ** vs. CG (NSD)
**Onan et al. (2023)** [28]	Telerehabilitation via videoconference	-Physical activity monitoring and personalized feedback -Tele-consultation with healthcare professionals -Therapeutic exercise program	8 w 3 d/w 45 min	Home Supervised	Supervised presential exercises	Pain (VAS): TG vs. CG (NSD) Neck Disability (NDI): TG vs. CG (NSD)
**Peterson et al. (2023)** [29]	Telerehabilitation via videoconference	-Physical activity monitoring and personalized feedback -Education in self-management skills -Therapeutic exercise program	4 w 4 d/w 20 min	Home Unsupervised	Supervised presential exercises	Pain (NRS): TG ** > CG (NSD) Neck Disability (NDI): TG ** > CG (NSD) General and physical health status (WHOQOL-BREF): TG ** vs. CG (NSD)

* *p* < 0.05; ** *p* < 0.001

## Data Availability

No new data were created or analyzed in this study. Data sharing is not applicable to this article.
